# Biological versus chronological ovarian age: implications for assisted reproductive technology

**DOI:** 10.1186/1477-7827-7-101

**Published:** 2009-09-22

**Authors:** Carlo Alviggi, Peter Humaidan, Colin M Howles, Donald Tredway, Stephen G Hillier

**Affiliations:** 1 Dipartimento di Scienze Ostetriche e Ginecologiche - Medicina della Riproduzione, Università degli Studi di Napoli Federico II, via S. Pansini 5, 80131 Naples, Italy; 2 The Fertility Clinic, Skive Regional Hospital, Skive, Denmark; 3 Merck Serono S.A. - Geneva (an affiliate of Merck KGaA, Darmstadt, Germany), Geneva, Switzerland; 4 Endocrinology and Reproductive Health GCDU, EMD Serono, Inc. (an affiliate of Merck KGaA, Darmstadt, Germany), Rockland, MA, USA; 5 University of Edinburgh, Centre for Reproductive Biology, Edinburgh, UK

## Abstract

**Background:**

Women have been able to delay childbearing since effective contraception became available in the 1960s. However, fertility decreases with increasing maternal age. A slow but steady decrease in fertility is observed in women aged between 30 and 35 years, which is followed by an accelerated decline among women aged over 35 years. A combination of delayed childbearing and reduced fecundity with increasing age has resulted in an increased number and proportion of women of greater than or equal to 35 years of age seeking assisted reproductive technology (ART) treatment.

**Methods:**

Literature searches supplemented with the authors' knowledge.

**Results:**

Despite major advances in medical technology, there is currently no ART treatment strategy that can fully compensate for the natural decline in fertility with increasing female age. Although chronological age is the most important predictor of ovarian response to follicle-stimulating hormone, the rate of reproductive ageing and ovarian sensitivity to gonadotrophins varies considerably among individuals. Both environmental and genetic factors contribute to depletion of the ovarian oocyte pool and reduction in oocyte quality. Thus, biological and chronological ovarian age are not always equivalent. Furthermore, biological age is more important than chronological age in predicting the outcome of ART. As older patients present increasingly for ART treatment, it will become more important to critically assess prognosis, counsel appropriately and optimize treatment strategies. Several genetic markers and biomarkers (such as anti-Müllerian hormone and the antral follicle count) are emerging that can identify women with accelerated biological ovarian ageing. Potential strategies for improving ovarian response include the use of luteinizing hormone (LH) and growth hormone (GH). When endogenous LH levels are heavily suppressed by gonadotrophin-releasing hormone analogues, LH supplementation may help to optimize treatment outcomes for women with biologically older ovaries. Exogenous GH may improve oocyte development and counteract the age-related decline of oocyte quality. The effects of GH may be mediated by insulin-like growth factor-I, which works synergistically with follicle-stimulating hormone on granulosa and theca cells.

**Conclusion:**

Patients with biologically older ovaries may benefit from a tailored approach based on individual patient characteristics. Among the most promising adjuvant therapies for improving ART outcomes in women of advanced reproductive age are the administration of exogenous LH or GH.

## Background

**Figure 1:**
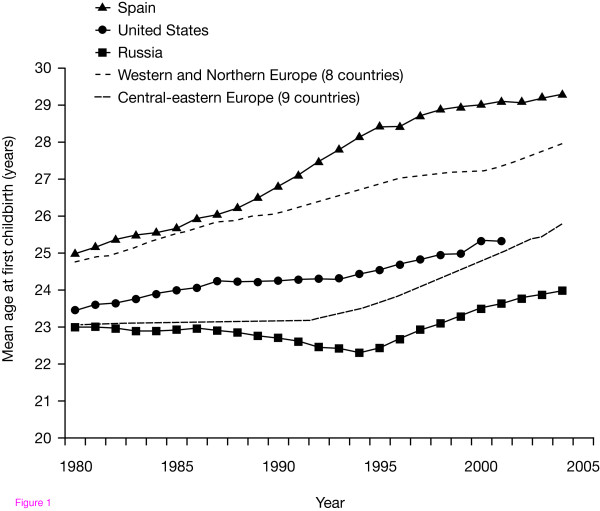
**Mean age at first childbirth in European women between 1980 and 2005**. Reproduced with permission from T. Sobotka (pers. commun.).

Over recent years, the average age of patients seeking infertility treatment has increased [1]. Since the development of effective contraception in the 1960s, women have been able to delay childbearing [2,3], and the average maternal age increased by approximately 5 years between the periods 1965-1969 and 1995-1999 [3]. The tendency towards deferred childbirth has also risen steadily since assisted reproductive technology (ART) treatments for infertility became available in 1980 [4,5] (Fig. 1).

After approximately 30 years of age, fertility decreases with increasing age, with a slow but steady decline in fertility in women aged between 30 and 35 years, which is followed by an accelerated decline [6,7]. Data from the 16^th ^to the early 19^th ^century show that women who married late were more likely to die childless; women who married when more than 35 years of age had twice the chance of dying childless compared with those who married when aged 30-34 years [8]. Thus, delayed childbearing reduces the chance of achieving a spontaneous pregnancy [6].

A combination of delayed childbearing and reduced natural fecundity with increasing age has resulted in a steady increase in the number and proportion of women aged ≥35 years who are seeking ART treatment [9]. Unfortunately, the outcomes of treatment with ART are also adversely affected by advanced patient age, and it is becoming increasingly important to optimize treatment outcomes for these older patients [10].

Although chronological age is the most important predictor of ovarian response to follicle-stimulating hormone (FSH), the rate of reproductive ageing varies considerably among individuals. Both environmental and genetic factors contribute to biological ovarian ageing. Thus, chronological and biological age are not always equivalent. We review here the principal developmental and endocrine mechanisms of ovarian ageing that underlie the variability between chronological and biological age. We will also discuss the effect of biological ovarian ageing on ART treatment outcomes and consider potential treatment strategies for those of advanced biological reproductive age.

## Methods

Electronic literature searches were performed via PubMed using combinations of the following keywords to identify relevant articles: 'AFC', 'age', 'AMH', 'ART', 'assisted reproduction', 'environmental', 'folliculogenesis', 'FSH', 'genetic', gonadotrophin', 'GH', 'infertility', 'LH', 'oogenesis' and 'ovarian'. All types of articles published in the English language were permitted and were unlimited by date of publication. The resulting publications were examined for relevance to the scope of the review (namely genetic and environmental factors that may influence ovarian ageing, the effect of biological ovarian ageing on ART outcomes, and potential treatment strategies for women of advanced biological reproductive age) and supplemented with other key publications that were known to the authors.

### Ovarian function: folliculogenesis and oogenesis

**Figure 2:**
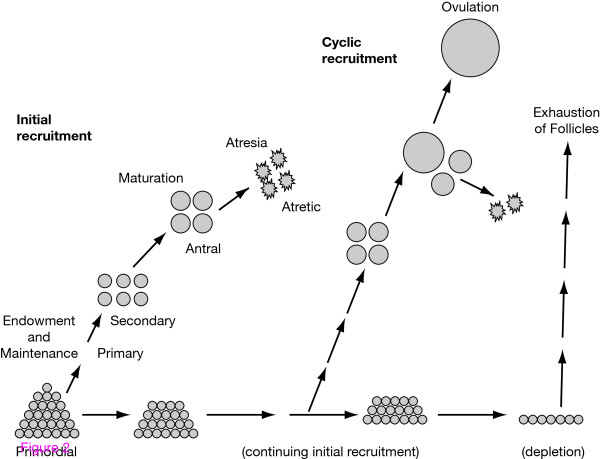
**Life history of ovarian follicles endowment and maintenance, initial recruitment, maturation, atresia or cyclic recruitment, ovulation, and exhaustion**. Reproduced with permission from McGee and Hsueh. Initial and cyclic recruitment of ovarian follicles. *Endocr Rev *2000, **21**:200-214. ^© ^2000. The Endocrine Society.

The different stages of folliculogenesis are illustrated in Fig. 2 [11]. At birth, the ovaries contain a stock of approximately 1-2 million oocytes that are arrested at the first meiotic prophase [12]. Oocytes at the first meiotic prophase undergo atresia or resume meiosis after activation by an ovulation-inducing luteinizing hormone (LH) surge to form the haploid gamete for fertilization. In fact, almost all (99.9%) of the oocytes present at birth undergo atresia via apoptotic mechanisms [13]. Indeed, the total number of viable oocytes present in the ovaries at the onset of puberty is a small fraction of that present at birth.

The life-cycle of preovulatory follicles can be broken down into three successive phases: initiation, which occurs from birth to old age and is independent of gonadotrophic support; FSH-dependent progression, which requires tonic stimulation by FSH; and LH-responsive maturation, which occurs when FSH-induced genes fall under LH control, leading to oestrogen secretion and ovulation [14,15]. These processes ensure that the 'right' number of follicles are available at the 'right' stage of development, at the 'right' time in the menstrual cycle.

#### Early stages

Early stages of follicular growth are characterized by oocyte enlargement, granulosa cell proliferation (forming multiple layers around the oocyte) and development of the theca interna. From early infancy onwards, the proportion of the total number of healthy primordial follicles that proceeds to further development remains constant throughout a woman's reproductive life. Therefore, as the number of oocytes present in the ovaries decreases, the absolute number of developing follicles progressively declines with age [16].

The mechanisms responsible for initiating follicular growth are independent of gonadotrophins and may originate in the oocyte itself [17]. When a primordial follicle grows, the oocyte increases in size and the flattened cells surrounding it become cuboidal and proliferate to form the granulosa cell layer; the structure is then called a 'primary' follicle. All primary follicles that develop before puberty are destined to undergo atresia because the gonadotrophin support needed to promote full preovulatory growth is absent until shortly after this event [18]. Granulosa cells in healthy primary follicles continue to divide to produce two or more layers while the zona pellucida forms around the enlarging oocyte. Outside the basement membrane, a layer of cells differentiates from the stroma and becomes the theca interna. The structure is now called a preantral follicle.

#### Intermediate stages

When approximately three layers of granulosa cells have formed in preantral follicles, fluid-filled spaces appear between the granulosa cells and gradually become confluent to form a single large antrum. Antrum formation is gonadotrophin-dependent. In humans, this growth stage lasts until follicles are approximately 2-4 mm in diameter. Healthy antral follicles are on the brink of entering the terminal stages of preovulatory development. However, to do so, they require appropriate, cyclical gonadotrophin stimulation. Thus, all antral follicles that develop before puberty will become atretic.

#### Ovulation

Usually one preovulatory follicle develops during each menstrual cycle, increasing in diameter from approximately 5 mm at the beginning of the cycle to more than 20 mm at ovulation 2 weeks later. During its last 6 days or so of development, a follicle increasingly secretes oestrogen and inhibin (INH), the classic biomarkers of preovulatory follicular development. These hormones lead to discharge of the mid-cycle LH surge from the pituitary gland, which in turn triggers resumption of oocyte meiotic maturation and ovulation.

### The mechanisms of ovarian ageing

#### Follicular loss and the menopause

As a woman ages, her fecundity declines because of the loss of follicles from the ovary [19] and an associated reduction in oocyte quality [20]. The reduction in oocyte quality is in line with the increased incidence of miscarriages and chromosomal aberrations that occur after the age of 35 years [21]. The number of follicles in the human ovary declines at a rate that is bi-exponential. The rate of follicle loss more than doubles when the numbers fall below the critical level of 25,000; which occurs at approximately 37.5 years of age [22].

It has been suggested that a threshold number of follicles is required to maintain a regular menstrual cycle [23]. The peri-menopausal period is characterized by increasing irregularity in cycle length. The transition from peri-menopause to menopause is complete when approximately 1000 follicles remain in the ovaries; this occurs at an average age of 51 years. The time taken to reach this point from the critical threshold of 25,000 follicles seems to be fairly constant, at approximately 13 years [24].

Several years before menstrual cycles cease, initiation of follicular growth begins to accelerate, speeding up the loss of the residual follicular stock [25]; this occurs at approximately 35 years of age. This effect is associated with a gradual increase in circulating levels of FSH.

#### The central role of FSH in termination of folliculogenesis

FSH orchestrates the termination of folliculogenesis in human ovaries. The increase in circulating plasma FSH levels appears to be due mainly to reduced secretion of follicular growth and differentiation factors related to transforming growth factor-b that negatively affect the release of FSH from the pituitary gland.

INH and anti-Müllerian hormone (AMH) are produced by immature ovarian follicles and help to regulate secretion of FSH by the pituitary gland [26,27]. As the number of immature follicles declines with age, negative feedback control on FSH secretion is relaxed, and basal circulating levels of FSH rise. High levels of FSH promote inappropriate maturation of the granulosa cells of the residual preantral (INH B-secreting) follicles containing ova that have not completed their gonadotrophin-independent growth phase. These follicles eventually become atretic, presumably because of the asynchronous maturation of their germinal and somatic components. The process is amplified as FSH levels continue to rise, until oestradiol and INH A levels also fall in the late peri-menopause, and menstrual cycles cease. The high FSH levels are also believed to underpin the gradual shortening of the follicular phase of the menstrual cycle with age, and the increased incidence of dizygotic twins [28].

### Variability in reproductive ageing

#### Variability in ovarian ageing

The size of the initial oocyte stock, the proportion that undergoes atresia and the rate of initiation of growth of follicles are genetically determined variables [16,29]. Thus, the age at which the menopause occurs varies among individuals, and is determined mainly by genetic factors.

The peri-menopausal period, from the onset of cycle irregularity to menopause, is reported to be approximately 6 years, regardless of the age at menopause [30]. Similarly, the onset of subfertility for each individual woman is believed to begin at a relatively fixed interval prior to the menopause. This is corroborated by data from ART studies: women who respond poorly to ovarian stimulation tend to reach the menopause earlier [31-34]. Women who respond poorly to ovarian stimulation may be at a stage between the accelerated decline and complete loss of fertility.

Epidemiological studies show that 10% of women in the general population are menopausal by the age of 45 years [35,36]. Assuming a time interval of 13 years between the accelerated decline of ovarian follicles and complete loss of fertility, a woman who reaches the menopause at the age of 45 years may have begun a decline in follicle quantity and quality at approximately 32 years of age (although the maintenance of regular menstrual cycles would make her unaware of this possibility). Therefore, 10% of women may be at risk of unexplained reduced fecundity during their third decade. These women may have a poor response to ovarian stimulation, and could be described as having early 'biological ovarian ageing'.

#### Variability in ageing reflected by levels of gonadotrophins

FSH levels begin to increase long before the onset of menstrual cycle irregularity, and continue to rise thereafter [37,38]. Levels of both FSH and LH rise steadily during the period of follicle depletion during the peri-menopausal period, but the detailed dynamics involved are incompletely understood. The serum LH concentration usually starts to increase after the rise in FSH concentration, but in some cases this effect is not observed at all [39-41].

Ferrell et al. conducted a 5-year prospective study in order to investigate age-related changes in LH and FSH, in a group of 156 women within an age range of 25-58 years at the beginning of the study [42]. The participants provided daily urine samples for 6 months per year in five consecutive years. These samples were analysed for aggregate distributions with age, individual trajectories with age, and variance in age-specific estimates within and between women.

**Figure 3:**
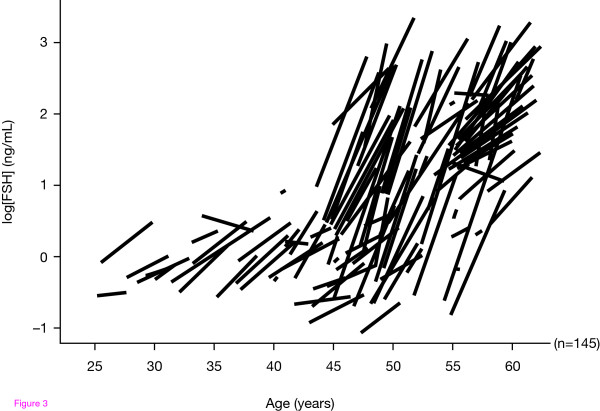
**Some women undergo premature menopause whereas others conceive naturally in their late forties**. Reproduced with permission from Ferrell et al. Monitoring reproductive aging in a 5-year prospective study: aggregate and individual changes in luteinizing hormone and follicle-stimulating hormone with age. *Menopause *2007, **14**:29-37.

The study confirmed that both FSH and LH levels increase with age, but the timing and magnitude of these changes were different for each hormone and varied among individuals. Both FSH and LH increased dramatically during the later peri-menopausal stages. FSH levels increased from normal to high within a relatively short time frame in the peri-menopausal years (<5 years). Although the most rapid increase in aggregate and individual FSH levels occurred after the age of 45, an increasing level of FSH was observed even in young women. However, any aggregate estimates of FSH or LH levels may be misleading as they represent a mixture of different trends seen in individuals, who have widely varying levels at different ages, and the rate of increase also differs among individuals. Figure 3 illustrates that the serum FSH rise started at different FSH concentrations for each woman. Thus, the findings of Ferrell et al. [42] illustrate the variable rate of ovarian ageing among individuals reflected in gonadotrophin levels. Moreover, independent of chronological age, ovarian ageing affects both oocyte fecundity and quality [43] and can negatively impact on the outcome of ART.

### Factors that influence the rate of ovarian ageing

Both genetic and environmental factors influence the rate of ovarian ageing and ovarian sensitivity to gonadotrophins.

#### Genetic influences on biological ovarian age and sensitivity to gonadotrophins

It is well established that genetic variations may affect ovarian response to gonadotrophins and ovarian ageing and even premature menopause. For instance, fragile X-associated primary ovarian insufficiency affects a proportion of female carriers of a mutated version of the *FMR1 *gene [44]. It has been suggested that certain single nucleotide polymorphisms also affect sensitivity to gonadotrophins and ovarian ageing [45].

An extreme example of the relevance of a genetic cause of reduced ovarian gonadotrophin sensitivity to accelerated biological ageing is provided by the FSH-receptor haplo-insufficient knock-out mouse (FSH-R+/-); these animals consequently have ovarian insensitivity due to a reduced number of FSH receptors [46]. FSH-R+/- mice reach reproductive maturity earlier but have smaller litter sizes than their wild-type counterparts [47]. There is no difference in the total number of follicles in the ovaries of 3-month-old FSH-R+/- versus wild-type mice. However, the ovaries of 7-month-old FSH-R+/- mice contain significantly fewer oocytes than wild-type controls, and serum gonadotrophins are higher [47].

Additionally, it has been demonstrated that oocytes obtained from older women may have inadequate reserves of energy due to age-related accumulated effects on their mitochondrial DNA [48]. Such a deficit results in mitochondrial dysfunction and a potential increase in the frequency of non-dysfunction, abnormalities in chromatid separation. These events probably contribute to the age-related increase in oocyte aneuploidy and the subsequent raised risk of having a foetus with a chromosome abnormality [49,50]. Another study suggests that chaotic mosaicism in human pre-implantation embryos is correlated with a low mitochondrial membrane potential, and this mechanism programs the developmental fate of embryos [51].

The importance of genetic characteristics in determining ovarian cycle and ovarian morphology with respect to the FSH receptor has also been described [52]. Women with the FSH receptor Ser^680^/Ser^680 ^genotype, comprising approximately 20% of the female population, showed a significant increase in total menstrual cycle length and time from luteolysis to ovulation compared with control subjects (Asn^680^/Asn^680 ^wild-type receptor). In addition, despite being normo-ovulatory, women with the Ser^680 ^polymorphism displayed a significantly higher serum FSH level compared with the control population [52]. Consequently, this genotype is associated with a higher ovarian threshold to FSH and women with the Ser^680 ^polymorphism have been reported to have a lower ovarian response to FSH stimulation during ART [53].

Although much research into ovarian resistance to gonadotrophins and ovarian ageing has focused on the gonadotrophin receptors, it is also important to consider the gonadotrophins themselves and other systems. A common variant of the LH gene (Trp^8^Arg and Ile^15^Thr of the beta subunit) encodes a protein with altered *in vitro *and *in vivo *activity [54], which thus may be less effective at supporting FSH-stimulated multi-follicular growth, resulting in a suboptimal ovarian response to standard stimulation regimens and in higher drug consumption [55]. An increased prevalence of the LH gene, beta subunit variant has been reported in Japanese patients with premature ovarian failure [56] and in Japanese infertility patients [57]. Therefore, women with this gene variant could benefit from exogenous LH supplementation during ovarian stimulation.

A possible link between ovarian age and the tumour suppressor gene, phosphatase and tensin homologue (PTEN) has been suggested. In a PTEN knock-out mouse model, all primordial ovarian follicles were activated prematurely [58]. In addition, although the low-density lipoprotein receptor-related protein 5 (*LRP5*) gene is considered to be primarily associated with bone metabolism via Wnt signalling, gene polymorphisms have been associated with a marked variation in circulating FSH levels in normal post-menopausal women [59].

#### Environmental influences on fecundity, biological ovarian age and sensitivity to gonadotrophins

Data from three epidemiological studies on the effect of biological and behavioural determinants of fertility in Bangladesh during 1975-1989 [60], 1981-1991 [61] and 1993-1994 [62] have shown that when women were stratified according to age there was a progressive decrease in fecundity rate, even among younger patients. Two studies reported that there was a significant effect of female education, female employment [61] and access to media (radio) on contraceptive use and a subsequent decline in fertility as women became more aware of family planning methods and viewed contraception more positively [62]. Lactational infecundity was also reported as a significant fertility-reducing factor in this population, although this factor was judged to remain constant while the fertility-reducing effect of contraception was predicted to increase [60].

Environmental factors may shorten the functional lifespan of a woman's ovaries. Diet may play a role in the occurrence of early menopause [63]. Cigarette smoking is one of the most common and important factors that has been found to reduce ovarian reserve [64]. Compounds in tobacco exert a deleterious effect on follicle maturation [65] and enhance follicle damage and premature ovarian failure [66]. The ovaries are extremely sensitive to aggressive chemotherapy or radiotherapy regimens for the treatment of cancer [67] and women receiving alkylating agents such as cyclophosphamide and chlorambucil are at particularly high risk of gonadal dysfunction [68]. Although the precise mechanisms involved are incompletely understood, ovarian histology studies have shown that follicle stores are depleted and ovarian atrophy occurs after chemotherapy or radiotherapy treatment [67,69-72].

#### The effect of co-existing pathology on ovarian sensitivity to gonadotrophins

There is an association between endometriosis and infertility (30-50% of patients with endometriosis are infertile) but the visible endometriotic lesions contribute only a small proportion of the reduced fecundity of these women [73]. Data suggest that the adverse effects of endometriosis are not only related to abnormalities of reproductive anatomy but rather act to impair oocyte development [74]. A meta-analysis of data from two randomized trials showed that laparoscopic excision or ablation of lesions may improve the fertility of women with mild or moderate endometriosis [75].

There is also evidence of reduced responsiveness to gonadotrophins following laparoscopic ovarian cystectomy [76], suggesting that surgical treatment of endometriosis may further decrease a woman's ovarian reserve. Thus, it has been suggested that ovarian surgery should be undertaken only for the treatment of large endometriotic cysts or to treat pain that is refractory to medical treatment or exclude malignancy [77].

### Impact of chronological versus biological age on ART treatment outcomes

**Figure 4:**
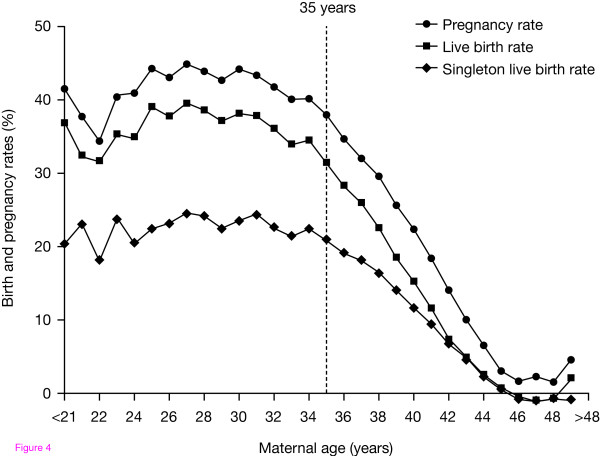
**Pregnancy and live birth rates following ART decline with increasing age**. Based on SART data.

Although ART treatments are now available for patients experiencing fertility problems, the likelihood of successful outcomes decreases with increasing female age. Poor ovarian response to stimulation is more common in women aged ≥35 years as the ovaries become less sensitive to FSH with increasing age [78]. The probability of embryo implantation and successful live birth after *in vitro *fertilization (IVF) also declines progressively in women over the age of 35 years [79,80] (Fig. 4). However, the outcome for patients using donor eggs remains relatively constant with increasing age, demonstrating that poor outcomes in older women relate to oocyte rather than to uterine factors [81].

Thus, biological ovarian age may be more important for the prognosis of fertility treatment than chronological ovarian age [43]. As a greater proportion of older patients present for ART, it will become increasingly important to critically assess their prognosis in order to counsel them appropriately and optimize treatment strategies. The use of biomarkers and genetic markers may allow estimation of the biological ovarian age of individual patients. This information could assist greatly in informing patients and optimizing their management.

#### Biological ovarian age and ovarian response

**Figure 5:**
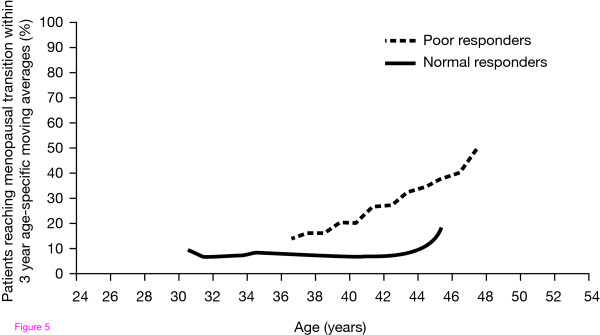
**Poor response is indicative of older biological age**. ***In vitro *fertilization poor response to follicle-stimulating hormone is associated with earlier age of menopause**. de Boer et al. Increased risk of early menopausal transition and natural menopause after poor response at first IVF treatment. *Hum Reprod *2003, **18**:1544-1552. Reproduced by permission of Oxford University Press.

As previously described, the menopause marks the depletion of follicles to below a critical threshold of approximately 1000, and the age at which menopause occurs is highly variable [24]. Biological ovarian age, as assessed by ovarian response to FSH during ART, can be used to predict the time to menopause (Fig. 5). A retrospective cohort study showed that a low residual number of oocytes, as reflected by a low number of oocytes (0-3) retrieved at first IVF treatment, is an important predictor of the risk of an early menopausal transition [82]. Ovarian response to gonadotrophins may, therefore, be important for appropriate counselling and management of patients undergoing ART.

#### Biological ovarian age and pregnancy rates

**Figure 6:**
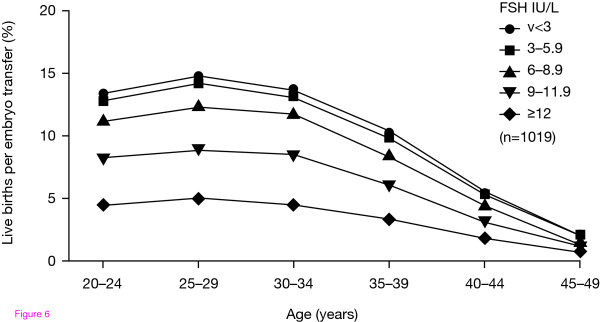
**Reduction in the rate of live births per embryo transferred against both increasing chronological age and increasing basal FSH in a prior spontaneous menstrual cycle**. Akande et al. Biological versus chronological ageing of oocytes, distinguishable by raised FSH levels in relation to the success of IVF treatment. *Hum Reprod *2002;**17**:2003-2008. Reproduced by permission of Oxford University Press. FSH, follicle-stimulating hormone.

Biological ovarian age as assessed by FSH levels is an important prognostic factor for IVF-related pregnancy rates (Fig. 6). Studies have shown that elevated basal FSH levels are associated with a poor response to ovarian stimulation, which may be due to reduced oocyte numbers and can lead to lower ART pregnancy rates [83-86]. Akande et al. studied the ability of fertilized oocytes to implant successfully in relation to ovarian age as indicated by basal FSH levels. The authors reported that ovarian ageing affects both oocyte fecundity and quality, and can occur independently of chronological age [43].

### ART management strategies for patients with biological ovarian ageing

Despite major advances in medical technology, there is currently no ART treatment strategy that can fully compensate for the natural decline of fertility with increasing age [2]. For example, simply using a high dose (300 IU) of FSH during ART will not improve pregnancy rates among women with an expected poor response (antral follicle count [AFC] [2-5 mm] < 5) [87]. Various adjuvant therapies for improving ART outcomes in women of advanced reproductive age have been suggested, such as the administration of exogenous LH or growth hormone (GH). Several potentially useful biomarkers are also emerging that can identify women with accelerated biological ovarian ageing. Patients with biologically older ovaries may benefit from a tailored approach based on individual characteristics to maximize the chances of the low numbers of healthy follicles developing successfully.

#### Assessment of biological age using biomarkers

Although the diagnostic accuracy and clinical value of currently available tests of ovarian reserve is modest [88], there has been much interest in the use of basal hormone levels (serum FSH, AMH, INH B) or the AFC prior to stimulation. The decreasing sensitivity of the ovaries to FSH with age is reflected by rising basal serum FSH levels, which provide a crude surrogate marker of biological ovarian age when paired with the chronological age.

The AFC represents a better marker than either chronological age or basal FSH for assessing the ovarian biological age, and may be used to select older patients who have a good prognosis for IVF [89]. Older patients undergoing ovarian stimulation for IVF generally have lower AFCs than younger patients. A high AFC gives an older patient a better prognosis for IVF treatment outcome [90-92]. However, different genetic characteristics with respect to the FSH receptor may mean that using AFC as a sole assessment of IVF prognosis may be confounded in some women. Women with the FSH receptor Ser^680^/Ser^680 ^genotype show a significantly greater increase in AFC during the early follicular phase compared with the Asn^680^/Asn^680 ^(wild-type) genotype, and Ser^680^/Ser^680 ^genotypes demonstrate ovarian resistance to FSH [52].

Because of their production by immature ovarian follicles, AMH and INH are also promising biomarkers of ovarian ageing [93,94]. Early follicular phase serum AMH levels correlate with the number of antral follicles [94-96] and oocytes retrieved [97,98] and live birth after ovarian stimulation [99]. AMH levels also decline progressively during the 5 years prior to the menopause [100].

Baseline AMH levels have been used successfully to individualize ovarian stimulation [101]. A recent study demonstrated that using a mixed treatment strategy of gonadotrophin-releasing hormone (GnRH) long agonist or antagonist protocols (rather than simply adjusting the FSH dose) resulted in good clinical pregnancy rates [101]. Importantly, it was shown that the use of a GnRH antagonist regimen for women with reduced circulating AMH levels (1-4.9 pmol/l) was associated with a trend towards increased clinical pregnancy rates, and reduced cycle cancellations and treatment burden compared with other study populations [101].

#### LH supplementation

The 'two cell - two gonadotrophin' model highlighted the role of LH in androgen production and release throughout folliculogenesis, and founded the concept that granulosa and theca cells are distinct follicular compartments regulated by FSH and LH, respectively [15,102]. This classic model has since been revised to take account of the induction of granulosa cell LH receptor expression by FSH during advanced stages of antral follicular development [15,103-105]. Thus, LH regulates and integrates both granulosa and theca cell function during late preovulatory development.

FSH and LH cooperate to induce the local production of growth factors, which are required for the paracrine regulation of follicular maturation (although LH supersedes FSH to sustain granulosa cell function during intermediate-late stages of folliculogenesis) [106,107]. It has been proposed that a lack of either gonadotrophin may be counteracted by higher levels of the other. Indeed, FSH activity can be totally substituted by LH once granulosa cells express adequate numbers of LH receptors [104,108]. Conversely, increasing the dose of exogenous FSH during controlled ovarian stimulation can overcome GnRH-agonist mediated reduction of LH in many women.

This hypothesis explains why a low LH concentration or activity (below a subcritical threshold) may lead to impaired granulosa paracrine signalling and a correspondingly higher requirement for FSH. Furthermore, this theory gave rise to a suggestion that women with ovarian resistance to exogenous FSH, such as those with biologically older ovaries, may benefit from LH supplementation. Although some studies have failed to show an effect of LH supplementation in older women (≥35 years [109,110] or ≥40 years [111]), evidence of a beneficial effect of LH supplementation is gradually accumulating.

In a prospective randomized study using a long GnRH down-regulation protocol, significantly lower implantation and pregnancy rates were observed in women aged ≥35 years than in those aged <35 years when only recombinant human (r-h)FSH was used for ovarian stimulation (*P *< 0.03 and *P *< 0.05, respectively); however, no age-related differences were found in women who received supplementation with r-hLH [112]. Moreover, the implantation rate among women aged ≥35 years who received r-hLH was significantly higher than in those who did not (*P *< 0.05) [112]. Similarly, the clinical pregnancy rate among patients aged ≥35 years at their first ART cycle was significantly higher if they received r-hLH than if they did not [113].

In a randomized controlled trial (using a GnRH antagonist), LH supplementation was associated with a significant improvement in implantation rates in women aged 36-39 years than stimulation with only FSH (26.7% vs 18.9%; *P *= 0.03), but this effect was not observed in younger patients [114]. Another positive effect of LH supplementation was reported in a study of women aged >37 years at risk of a poor ovarian response (FSH > 7.6 IU/l) who received a flexible GnRH antagonist protocol with r-hLH supplementation, which resulted in a significantly greater number of mature oocytes than did a standard GnRH agonist short protocol [115].

Recent studies have attempted to define specific patient populations that would benefit most from LH supplementation. The greatest benefit of LH in ART has been seen in women with reduced responsiveness to gonadotrophin stimulation [116-121], which, it could be hypothesized, are those with biologically older ovaries. These early data are supported by a Cochrane systematic review in which a significantly higher pooled estimate of ongoing pregnancy was demonstrated in a sub-analysis of data from poor responders who received r-hLH supplementation [116].

LH could improve treatment outcomes following ART without affecting oocyte numbers, for example via beneficial effects of LH on oocytes via indirect routes such as cumulus cells [122]. Cumulus cells play a vital role in the maturation of oocytes during folliculogenesis. The direct action of LH may protect cumulus cells from apoptosis, so that they can continue to support the developing oocyte until ovulation, and ensure that the oocyte can sustain fertilization and early phases of embryogenesis [122].

Given the conflicting evidence and debate, more randomized-controlled trials are required to fully explore the potential utility of LH supplementation in IVF protocols.

#### GH supplementation

mRNA transcripts for the GH receptor are expressed in human oocytes and throughout pre-implantation embryonic development [123,124]. Indeed, human GH (hGH) receptors are present in cumulus cells, and both immature germinal vesicle (GV) and mature metaphase II stage oocytes [124]. Low concentrations of hGH in follicular fluid are associated with total fertilization failure, cleavage failure and poor embryo morphology [125].

It is likely that GH plays a role in the final stages of human oocyte maturation and early embryogenesis as it does for several other mammalian species [124]. hGH stimulates cytoplasmic maturation and may have a positive role in increasing total cell number in the embryo and in decreasing apoptosis [126], as described in bovine species [127]. In addition, GH is reported to upregulate the synthesis of insulin-like growth factor (IGF)-I, which acts to amplify the effects of FSH on granulosa and theca cells [128].

These observations suggested a possible role for hGH in *in vitro *oocyte maturation during ART. Early studies have confirmed that the addition of hGH to culture medium improves *in vitro *maturation of immature GV oocytes [129,130]. Thus, the *in vitro *use of hGH may offer an opportunity to rescue cycles in which a high proportion of immature oocytes are retrieved [130].

The use of hGH in the management of female subfertility was first reported in the early 1990s [131,132] and since then its clinical use has been controversial. Although there are substantial *in vitro *data showing the critical importance of the IGF-IGFBP family (IGF-I, IGF-II and their binding proteins) to follicular development [133,134], subsequent studies failed to demonstrate a therapeutic advantage [135-141].

Interestingly, recent studies have shown more promising results. In a randomized trial of 100 women aged >40 years, co-stimulation with hGH (8 IU daily) led to significantly higher plasma and intrafollicular oestradiol levels, and clinical pregnancy and live birth rates, than did a standard ovarian stimulation protocol [142]. These data suggest that hGH may improve the potential for oocyte development and counteract the age-related decline of oocyte quality [142]. Kucuk et al. reported a significant increase in the number of oocytes recovered in poor responders receiving GH co-treatment with a GnRH agonist long protocol [128]. A recent systematic review and meta-analysis on the use of hGH in poor responders to ovarian stimulation concluded that there is some evidence to support a beneficial effect of GH on pregnancy rates [143]. Four placebo-controlled studies of poor responders were identified [131,136,139,144] and data from 82 patients were pooled. The addition of GH to ovarian stimulation protocols was shown to significantly increase live birth rates (odds ratio 5.22, 95% confidence interval 1.09-24.99; *P *= 0.04).

## Conclusion

Biological ovarian age is more important than chronological age in predicting the outcome of ART. An increasing number of older patients are now presenting for ART treatment, and efforts should be made to critically assess each patient's biological ovarian age in order to counsel them appropriately regarding prognosis, and optimize individual treatment. When endogenous LH levels are heavily suppressed by GnRH analogues, LH supplementation may help to optimize treatment outcomes for women with biologically older ovaries. The potential genetic influences on ovarian gonadotrophin sensitivity discussed here are intriguing, and have encouraged an approach based on systems biology to further improve our understanding of gonadal function and ageing. Such translational medical research could refine the use of current therapies, producing valuable predictive models to guide use of current treatments, and may ultimately lead to a range of new therapies.

## Competing interests

CA, SH and PH have declared no conflicts of interest. CMH is an employee of Merck Serono S.A. - Geneva, Switzerland (an affiliate of Merck KGaA, Darmstadt, Germany). DT was an employee of EMD Serono, Inc., Rockland, MA, USA (an affiliate of Merck KGaA, Darmstadt, Germany) when the manuscript was in development.

## Authors' contributions

All authors were involved with the design, writing and reviewing of this manuscript and have approved the final version for submission.
